# Prognosis of breast cancer molecular subtypes in routine clinical care: A large prospective cohort study

**DOI:** 10.1186/s12885-016-2766-3

**Published:** 2016-09-15

**Authors:** André Hennigs, Fabian Riedel, Adam Gondos, Peter Sinn, Peter Schirmacher, Frederik Marmé, Dirk Jäger, Hans-Ulrich Kauczor, Anne Stieber, Katja Lindel, Jürgen Debus, Michael Golatta, Florian Schütz, Christof Sohn, Jörg Heil, Andreas Schneeweiss

**Affiliations:** 1 Department of Gynecology and Obstetrics, University of Heidelberg, Im Neuenheimer Feld 440, 69120 Heidelberg, Germany; 2 Division of Clinical Epidemiology and Aging Research, German Cancer Research Center (DKFZ), Im Neuenheimer Feld 581, 69120 Heidelberg, Germany; 3 Department of Pathology, University of Heidelberg, Im Neuenheimer Feld 224, 69120 Heidelberg, Germany; 4 National Center for Tumor Diseases (NCT), University Hospital, Im Neuenheimer Feld 460, 69120 Heidelberg, Germany; 5 Radiology Department, University of Heidelberg, Im Neuenheimer Feld 400, 69120 Heidelberg, Germany; 6 Department of Radiation Oncology, University of Heidelberg, Heidelberg, Germany

**Keywords:** Breast cancer, Molecular subtypes, Outcome, Breast care unit

## Abstract

**Background:**

In Germany, most breast cancer patients are treated in specialized breast cancer units (BCU), which are certified, and routinely monitored. Herein, we evaluate up-to-date oncological outcome of breast cancer (BC) molecular subtypes in routine clinical care of a specialized BCU.

**Methods:**

The study was a prospectively single-center cohort study of 4102 female cases with primary, unilateral, non-metastatic breast cancer treated between 01 January 2003 and 31 December 2012. The five routinely used molecular subtypes (Luminal A-like, Luminal B/HER2 negative-like, Luminal B/HER2 positive-like, HER2-type, Triple negative) were analyzed. The median follow-up time of the whole cohort was 55 months. We calculated estimates for local control rate (LCR), disease-free survival (DFS), distant disease-free survival (DDFS), overall survival (OS), and relative overall survival (ROS).

**Results:**

Luminal A-like tumors were the most frequent (44.7 %) and showed the best outcome with LCR of 99.1 % (95 % CI 98.5; 99.7), OS of 95.1 % (95 % CI 93.7; 96.5), and ROS of 100.0 % (95 % CI 98.5; 101.5). Triple negative tumors (12.3 %) presented the poorest outcome with LCR of 89.6 % (95 % CI 85.8; 93.4), OS of 78.5 % (95 % CI 73.8; 83.3), and ROS of 80.1 % (95 % CI 73.8; 83.2).

**Conclusions:**

Patients with a favorable subtype can expect an OS above 95 % and an LCR of almost 100 % over 5 years. On the other hand the outcome of patients with HER2 and Triple negative subtypes remains poor, thus necessitating more intensified research and care.

**Electronic supplementary material:**

The online version of this article (doi:10.1186/s12885-016-2766-3) contains supplementary material, which is available to authorized users.

## Background

Breast cancer (BC) mortality has declined over the past decade in most developed countries, due to new developments in screening, diagnostics, surgery, radiotherapy, and (neo) adjuvant systemic therapy, in conjunction with structural improvements (multidisciplinarity, implementation of specialized breast cancer units) and target agreements (evidence-based guidelines, certification processes) [1, 2]. Over the past decade, increasing molecular and genetic knowledge [3–6] has provided a new understanding of breast cancer as a heterogeneous, systemic disease that can be classified into different subtypes with different clinical and pathological features, different therapeutic response patterns, and different outcomes [7, 8]. The main molecular classification of breast cancer have been distinguished by gene expression profiling into intrinsic subtypes by Peru et at [5]. These modern microarray-based gene expression profiles (GEP) are the best way to visualize the heterogeneity of breast cancer, but lacking gene expression profiling in clinical routine due to cost and practicability made a surrogate classification necessary [9]. The molecular subtypes of breast cancer correspond reasonably well to a clinical characterization on the basis of hormone-and HER2 status, as well as proliferation markers or histological grade [10]. So the classification based on immunohistochemistry (IHC) markers was recommended by the St. Gallen Expert Consensus in 2011 [11] and confirmed again in 2013 [12]. It has become the accepted standard in routine clinical patient care. Classification into five molecular subtypes (Luminal A-like, Luminal B/HER2 negative-like, Luminal B/HER2 positive-like, HER2-type, Triple negative) helps to sort patients into groups with divergent prognoses and different response patterns to specific Every-day-routine outcome assessment of specialized breast cancer unit (BCU) must validate guideline-based care of BC patients in order to optimize the therapy of every individual case. This paper reports the outcome data of a prospective cohort of 4102 patients with primary, unilateral, non-metastatic BC treated at a specialized BCU according to routinely used molecular subtype definitions based on immunohistochemistry markers.

## Methods

### Patients

Since 01 January 2003 the medical history and the demographic, diagnostic, therapeutic, and follow-up data of all breast cancer patients referred to the BCU at Heidelberg University have been prospectively entered into our database. This register is routinely used for certification purposes and is monitored.

Patients from the registry were included in the present analysis if they had invasive or carcinoma-in-situ cancer of the breast and were newly diagnosed or treated between 01 January 2003 and 31 December 2012.

Patients were excluded from this analysis for any of the following reasons: male sex (*n =* 38), distant metastasis at the intake visit (M1, *n =* 296) or bilateral tumors (*n =* 619).

Patients with incomplete immunohistological information (149, i.e. 4.1 % of 3603) were included in the overall analysis, but they could not be considered in the subgroup outcome analysis.

### Histology and stage

Tumors were defined according to the World Health Organization [13], graded along Elston and Ellis [14], and grouped into stages according to the TNM classification [15]. The expression of estrogen receptor (ER), progesterone receptor (PR), human epidermal growth factor receptor 2 (HER2), and Ki-67 were assessed with an IHC assay of formalin-fixed, paraffin-embedded tumor tissue according to international standards.

### Subgroups

According to the St. Gallen International Expert Consensus recommendations 2011 [11], five molecular subtypes of invasive breast cancer have been differentiated by their expression of the IHC markers ER, PR, HER2, and Ki-67:

**Table:** 

**Intrinsic suptype**		**ER and/or PR**	**HER2**	**Ki-67**
Luminal A-like	(LumA)	+	−	<14 %
Luminal B/HER2 negative-like	(LumB/HER2 neg.)	+	−	≥14 %
Luminal B/HER2 positive-like	(LumB/HER2 pos.)	+	+	any
HER2-type	(HER2)	both−	+	any
Triple negative	(TN)	both−	−	any

The classification of 2011 was used because it corresponded best to the way we had categorized patients during the time period covered in this report [11].

Positivity for ER and PR was defined as an Immunoreactive Score [16] of at least 1 out of 12 or a Total Score [17] of at least 1 out of 8. All cases of non-invasive carcinoma-in-situ (CIS, regardless of specific subtype) have been defined as an additional subgroup for a separate analysis.

For invasive BC, the cell proliferation marker Ki-67 was available in the majority of our cohort (3004/3603, 83.4 %), while grading, either 1 or 3, was used in 599 of the 3603 cases (16.6 %) for subgroup classification (Table 1). For the differentiation of Luminal-like tumors, cases with a negative HER2 receptor status in combination with a positive ER or PR receptor and a grading of 1 led to the attribution of the Luminal A-like subgroup. In contrast a grading of G3 was assigned to the subgroup of Luminal B/HER2 negative-like tumors.

**Table 1:** Patient, tumor, and surgical therapy characteristics of all female cases with primary, non-metastatic, unilateral breast cancer diagnosed at the Heidelberg Breast Care Unit between 01 January 2003 and 31 December 2012

Total cases (*n =* 4102)	Number of cases	Percent (%)
Patient characteristics		
Age at diagnosis in years (*n =* 4102)		
median	57 years	
< 51	1355	33.0
51–65	1638	39.9
> 65	1109	27.0
total	4102	100.0
Menopausal status (*n =* 4102)		
pre	1377	33.6
peri	130	3.2
post	2498	60.9
missing	97	2.4
total	4102	100.0
Affected breast (*n =* 4102)		
left	2066	50.4
right	2036	49.6
total	4102	100.0
Tumor characteristics		
Main tumor histology (*n =* 4102)		
In-situ Carcinoma	499	12.2
Invasive Carcinoma	3603	87.8
Invasive ductal carcinoma (no specific type)	3082	85.5
Invasive lobular carcinoma	481	13.3
other (e.g. invasive medullar/mixed)	40	1.1
total	4102	100.0
T stage for invasive cases with adjuvant therapy (*n =* 2997)		
pT1	1863	62.2
pT1a	161	
pT1b	486	
pT1c	1202	
pTmic	7	
unknown	7	
pT2	909	30.3
pT3	138	4.6
pT4	58	1.9
pTx/missing	29	1.0
total	2997	100.0
T stage for invasive cases with neoadjuvant therapy (*n =* 606)		
ypT0	168	27.7
ypTis	16	2.6
ypT1	224	37.0
ypT1a	47	
ypT1b	52	
ypT1c	115	
ypTmic	8	
unknown	2	
ypT2	127	21.0
ypT3	47	7.8
ypT4	16	2.6
ypTx/missing	8	1.3
total	606	100.0
N stage for invasive cases (*n =* 3603)		
pN0	2473	68.6
pN1	655	18.2
pN2	243	6.7
pN3	158	4.4
pNx/missing	74	2.1
total	3603	100.0
Grading (invasive cases, *n =* 3603)		
Grade 1	600	16.7
Grade 2	1924	53.4
Grade 3	962	26.7
missing	117	3.2
total	3603	100.0
Estrogen receptor (invasive cases, *n =* 3603)		
positive	2877	79.9
negative	585	16.2
missing	141	3.9
total	3603	100.0
Progesterone receptor (invasive cases, *n =* 3603)		
positive	2599	72.1
negative	859	23.8
missing	145	4.0
total	3603	100.0
HER2 receptor (invasive cases, *n =* 3603)		
positive	346	9.6
negative	3118	86.5
missing	139	3.9
total	3603	100.0
Ki-67 status (invasive cases, *n =* 3603)		
< 14 %	1463	40.6
≥ 14 %	1541	42.8
missing	599	16.6
total	3603	100.0
Surgical therapy characteristics		
Surgical therapy (*n =* 4102)		
Breast Conserving Surgery	2999	73.1
Mastectomy	1103	26.9
total	4102	100.0
Axillary staging (*n =* 4102)		
SLND only	1671	40.7
SLND + ALND	501	12.2
ALND only	1600	39.0
none	330	8.1
total	4102	100.0

*SLND* sentinel lymphadenectomy, *ALND* axillary lymphadenectomy

### Treatment

The Heidelberg University BCU was fully certified on 10 October 2003, by the German certification board of the German Cancer Society and the German Society for Senology on the basis of the management of cases in 2002 and 2003. Thus all the cases included in this study were managed under certified conditions, which were confirmed by an annual re-certification process [18, 19].

### Endpoints and outcome assessment

The outcome from the time of diagnosis was assessed for the whole cohort, the five BC subtypes, and the CIS cases for several outcome parameters. The endpoints were local control rate (LCR), disease-free survival (DFS), distant disease-free survival (DDFS), overall survival (OS), and relative overall survival (ROS). Relative survival was defined as the ratio of the observed survival to the survival expected in the general West German population of the same age and sex during the same period of time [20].

Outcome was assessed as follows. First, hospital records were reviewed to obtain information with regard to survival, local and regional relapse, and distant metastasis. If outcome information was not available in the hospital record, the patient’s family doctor or gynecologist was contacted by mail or phone. If the required information could not be obtained by this approach either, an inquiry about the patient’s survival status was made at the responsible residents’ registration office. If the patient was still alive, she was contacted by mail and asked whether she had developed local or distant relapse with a detailed questionnaire. Follow-up was performed for cases diagnosed until 31 December 2012. Within this study period (starting 01 January 2003) *n =* 2322 patients had a complete follow-up information (i.e. could be followed until either death or study end. Of the remaining 1780 patients, 140 were lost to follow-up during the years 2003–2011, i.e. in total 140/4102 (3.4 %). The median time of follow-up was 45 months among those who were lost to follow-up, slightly shorter than among the whole cohort (55 months).

### Statistical analysis

The data were analyzed using SAS software version 9.3 (SAS Institute Inc.; Cary, NC, USA) and SPSS software version 22 (IBM; Armonk, NY, USA). The proportions of patients experiencing events at 5 years, the corresponding 95 % confidence intervals (95 % CI), and all survival plots are based on Kaplan-Meier estimates using PROC LIFETEST with the actuarial approach. Relative survival rates at 5 years were also calculated. The expected survival of the general population was calculated according to the Ederer II method [21], based on life tables for Germany for the years 2002 to 2010.

## Results

### Patient characteristics

The final cohort comprised 4102 patients, of which, 3603 (87.8 %) had invasive carcinoma and 499 (12.2 %) had CIS. Most invasive carcinoma cases were hormone receptor positive (ER: 79.9 %, PR: 72.1 %), HER2 negative (86.5 %), and had a grading of 2 (53.4 %). Most of the patients had a maximum tumor size of 2 cm (pT1: 62.2 %) without axillary lymph node involvement (pN0 68.6 %). Median age of the whole cohort was 57 years and most patients were postmenopausal (60.9 %). Breast conservation surgery was performed in 73.1 % of the study cohort and a mastectomy in 26.9 %. Concerning surgical management of the axilla sentinel lymph node biopsy alone (SLND) was performed in 40.7 % and axillary lymph node dissection in 39.0 % of the patients. Detailed patient characteristics of the cohort are shown in Table 1.

The UICC stage distribution (Additional file 1: Table S4) as well as the frequency of age, menopausal status and laterality (Additional file 2: Table S5) for the different subtypes can be found in the supplementary material.

### Outcome analysis

**Fig. 1:**
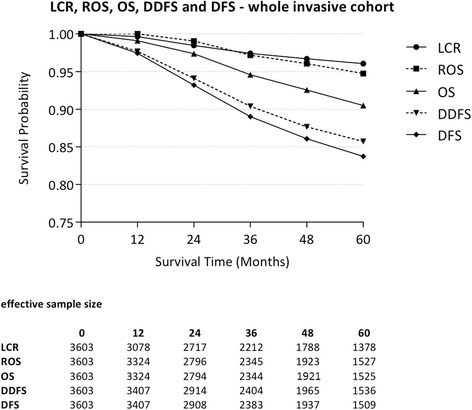
LCR, ROS, OS, DDFS and DFS-whole invasive cohort. Kaplan-Meier survival plot for LCR, ROS, OS, DDFS, and DFS for the cohort of invasive cases. Shown are annual survival rates. The table presents the effective sample size for each interval. [LCR: local recurrence rate; DFS: disease-free survival; DDFS: distant disease-free survival; OS: observed overall survival; ROS: relative overall survival]

**Fig. 2:**
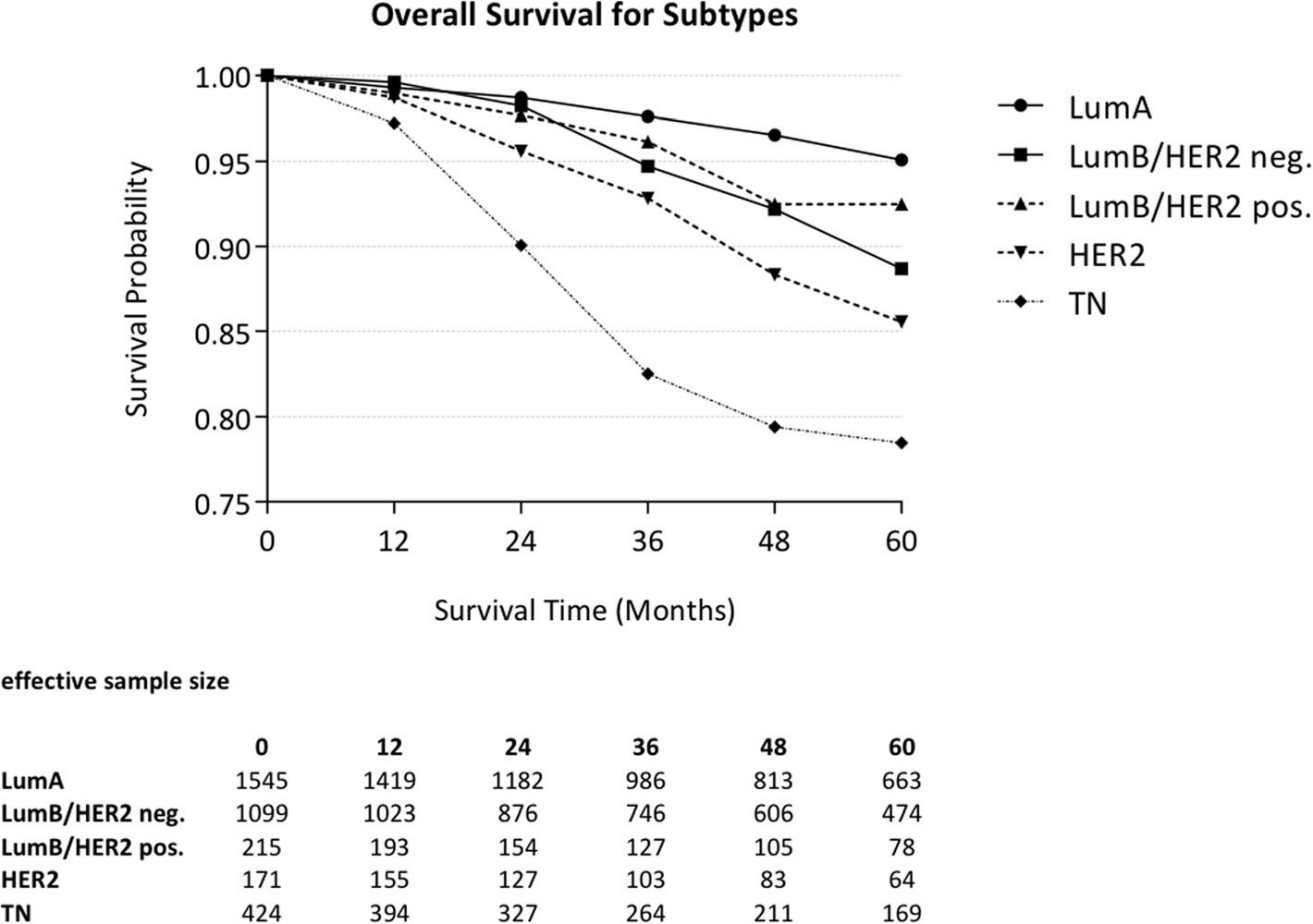
Overall Survival for Subtypes. Kaplan-Meier survival plot for overall survival according to molecular subtype (of invasive cancer). The annual survival rates for the following subtypes are shown: LumA, LumB/HER2 neg., LumB/HER2 pos., HER2, and Triple negative (TN). The table presents the effective sample size for each interval

For all patients with invasive disease, LCR was 96.1 % (95 % CI 95.3; 96.9); DFS was 83.7 % (95 % CI 82.2; 85.2); DDFS was 85.7 % (95 % CI 84.3; 87.1); OS was 90.5 % (95 % CI 89.3; 91.7) and ROS was 97.7 % (95 % CI 93.4; 96.0) at 5 years. As regards cancer subtypes, 44.7 % were luminal A-like, 31.8 % Luminal B/HER2 negative-like, 6.2 % Luminal B/HER2 positive-like, 5.0 % HER2-type, and 12.3 % Triple negative. The Luminal A-like subtype showed the best outcome: LCR was 99.1 % (95 % CI 98.5; 99.7); DFS was 92.1 (95 % CI 90.5; 93.9); DDFS was 92.9 % (95 % CI 91.3; 94.5); OS was 95.1 % (95 % CI 93.7; 96.5) and ROS was 100.0 % (95 % CI 98.5; 101.5). The Triple negative subtype had the worst outcome: LCR at 5 years was 89.6 % (95 % CI 85.8; 93.4); DFS was 69.1 % (95 % CI 64.1; 74.1); DDFS was 72.2 % (95 % CI 67.3; 77.1); OS was 78.5 % (95 % CI 73.8; 83.2); and ROS was 80.1 % (95 % CI 75.1; 85.1). Outcome measures for the whole cohort, with or without inclusion of CIS cases, and for all clinico-pathological subtypes at 5 years are presented in Tables 2 and 3. The corresponding Kaplan-Meier plots are shown in Figs. 1 and 2.

**Table 2:** Five-year outcomes of 5 different endpoints for all female patients with primary, non-metastatic, unilateral breast cancer treated at the Heidelberg Breast Care Unit between 01 January 2003 and 31 December 2012

	All patients (including in-situ) *n =* 4102 (including 499 in-situ cases)	Patients with invasive cancer (excluding in-situ) *n =* 3603
LCR [%] (95 % CI)	96.1 (95.3; 96.9)	96.1 (95.3; 96.9)
DFS [%] (95 % CI)	84.9 (83.6; 86.2)	83.7 (82.2; 85.2)
DDFS [%] (95 % CI)	86.9 (85.7; 88.1)	85.7 (84.3; 87.1)
OS [%] (95 % CI)	91.3 (90.2; 92.4)	90.5 (89.3; 91.7)
ROS [%] (95 % CI)	95.5 (94.3; 96.7)	94.7 (93.4; 96.0)

*CI* confidence interval, *LCR* local recurrence rate, *DFS* disease-free survival, *DDFS* distant disease-free survival, *OS* observed overall survival, *ROS* relative overall survival

**Table 3:** Outcome results of 5 different endpoints for all female cases with primary, non-metastatic, unilateral breast cancer treated at the Heidelberg Breast Care Unit between 01 January 2003 and 31 December 2012, (for whom all necessary histological information were available for distinct subtype attribution), differentiated by the invasive clinico-pathological tumor subtype or in-situ tumor (CIS). Results in percent at 5 years (95 % CI)

	INVASIVE CANCER					CIS
	LumA-like	LumB/HER2 neg.-like	LumB/HER2 pos.-like	HER2-type	Triple negative	
	*n =* 3454 (100 %) [missing due failed distinct subtype distribution *n =* 149, i.e. 4.1 % of invasive cohort]					*n =* 499 (100 %)
	*n =* 1545	*n =* 1099	*n =* 215	*n =* 171	*n =* 424	
	44.7 %	31.8 %	6.2 %	5.0 %	12.3 %	
LCR [%] (95 % CI)	99.1 (98.5; 99.7)	95.2 (93.6; 96.8)	95.0 (91.3; 98.7)	90.5 (84.7; 96.3)	89.6 (85.8; 93.4)	96.2 (93.9; 98.5)
DFS [%] (95 % CI)	92.2 (90.5; 93.9)	80.1 (77.2; 83.0)	79.0 (71.9; 86.1)	77.0 (69.4; 84.6)	69.1 (64.1; 74.1)	93.0 (90.2; 95.8)
DDFS [%] (95 % CI)	92.9 (91.3; 94.5)	82.2 (79.5; 84.9)	82.8 (76.0; 89.6)	83.3 (76.6; 90.0)	72.2 (67.3; 77.1)	95.6 (93.5; 97.1)
OS [%] (95 % CI)	95.1 (93.7; 96.5)	88.7 (86.2; 91.2)	92.5 (87.9; 97.1)	85.6 (78.6; 92.6)	78.5 (73.8; 83.2)	96.9 (94.8; 99.0)
ROS [%] (95 % CI)	100.0 (98.5; 101.5)	93.4 (90.7; 96.1)	96.0 (91.2; 100.8)	88.8 (81.5; 96.1)	80.1 (75.1; 85.1)	100.8 (98.6; 103.0)

*CI* confidence interval, *LCR* local recurrence rate, *DFS* disease-free survival, *DDFS* distant disease-free survival, *OS* observed overall survival, *ROS* relative overall survival, *CIS* carcinoma-in-situ

Additional outcome analyses for subtypes subdivided into UICC stages I-IIa (Additional file 3: Table S6) can be found in the supplementary material.

## Discussion

The 5-year OS for all patients with primary invasive breast cancer was 90.5 % (95 % CI 89.3; 91.7), and the ROS was 94.7 % (95 % CI 93.4; 96.0) (Table 2). This confirms the favorable prognosis of primary non-metastatic breast cancer receiving adequate treatment. In this study, we focused on a well-defined and homogenous patient cohort. The outcomes seen here can be expected at any specialized BCU. Most of the outcomes statistics published in the literature derive from clinical trials with the exclusion of certain types of patients commonly seen in routine care, e.g. the elderly patients with comorbid conditions. Thus, it is important to assess outcomes among a complete, unselected patient population seen in a routine clinical setting. On the basis of the favorable outcome results reported here, additional quality-of-life aspects might be brought more into focus for outcome quality for specific subgroups in the future.

In the face of unavailable gene expression profiles in clinical routine, the BC surrogate classification according to the St. Gallen Consensus 2011 [11] allows a differentiation of five molecular subtypes with distinct prognoses. Although the management of BC patients according to these subtypes has gained importance, it is beyond controversy that the traditionally assessed tumor characteristics, e.g. nodal status and tumor size, still have independent prognostic impact [22]. Because the St. Gallen subtype classification is widely accepted as a surrogate for subtyping according to intrinsic signatures [9], we used this classification for subtype-specific outcome analysis as they are distinct and well applicable in the context of outcome assessment. Standard pathological assessments seem adequate to define useful groups such as TN, HER2-type, and LumB/HER2 pos.-like tumors, for which treatment recommendations are seldom controversial [23]. In contrast to other studies (e.g. [24]), the Ki-67 score was available for the vast majority of cases, enabling us to differentiate the Luminal-like HER2 negative tumors. Nevertheless, the validity and robustness of Ki-67 is still controversial, although it has been widely accepted as a cell proliferation marker that is widely available [25]. Especially the St. Gallen 2011 cut-off recommendation of 14 % for Ki-67 (which was proposed and validated by Cheang et al. [26]) has been viewed critically due to a substantial inter-observer and intra-observer variability, especially for mid-range Ki-67 scores [27–29]. This discordance is highly problematic because a recommendation for or against chemotherapy for hormone receptor positive, HER2 negative, grade 2 tumors depends mainly on the Ki-67 threshold in the St. Gallen Consensus 2011.

Because of this ambiguity in defining exact surrogate subtypes it might be difficult to compare subtype outcome results with other studies that used different surrogate definitions. Despite this difficulty in comparison with other study designs the general trend concerning distribution (at least for clear defined subtypes like TN) and outcome in our cohort is in approximate accordance with other results e.g. from Canada [30], USA [31, 32], South Korea [33], Belgium [24], Spain [34, 35], Italy [36] and France [37]: LumA and LumB tumors were the most frequent (LumA was 44.7 %, LumB/HER2 neg. was 31.8 %, and LumB/HER2 pos. was 6.2 %), followed by TN cancers (12.3 %) and HER2 type (5.0 %). For the majority of patients with a Luminal A type a very favorable OS over 5 years of 95.1 % (95 % CI 93.7; 95.5) and an excellent LCR of 99.1 % (95 % CI 98.5; 99.7) was possible. But it becomes also evident that outcome possibilities for HER-2 type and TN cases are still much poorer even in times of more effective systemic treatment (Table 3). Two exemplary studies with large cohorts-the single-hospital report from Broukhaert et al. in Belgium [24] and the population-based report from Minicozzi et al. in Italy [36]-both used similar criteria approximating the St. Gallen 2011 classification. These two studies had quite comparable distributions of BC subtypes (42 % and 56 % for LumA-like, 27 % and 22 % for LumB/HER2 neg.-like, 14 % and 7 % for LumB/HER2 pos.-like, 7 % and 4 % for HER2-type, and 11 % and 10 % for TN). And these two studies also found similar outcomes; the DFS over 5 years was 93.0 % and 94.6 % for LumA-like, 87.4 % and 85.7 % for Lum B/HER2 neg.-like, 86.3 % and 86.8 % for LumB/HER2 pos.-like, 77.9 % and 79.7 % for HER2-type, and 80.5 % and 81.0 % for TN. DFS was somewhat lower for LumB-like and TN in our cohort than in those two other studies. Besides slightly different subtype and endpoint definitions, it must be considered that Broukhaert et al. used tumor grade instead of Ki-67 for defining subtypes, (with the associated problems mentioned above), and Minicozzi et al. studied a retrospective cohort (2003–2005) with a different Ki-67 cut-off and lack of reliable information about how Ki-67 was determined at that time.

In our cohort the median age of early breast cancer patients was 57 years compared to 64 years in Germany. Concerning surgical procedures mastectomy was performed in 26.9 % of all patients, which is lower than in a current published report from the SEER database with a mastectomy rate of 34 % showing an increase in the United States especially in women with node-negative and in-situ disease (Table 1) [38]. The widely use of specific anti-HER2 therapy in this cohort could increase outcome for patients with HER2 positive breast cancer [39]. The subgroup of patients with Luminal B/HER2 positive-like reveals a LCR of 95.0 % (95 % CI 91.3; 98.7) and a ROS of 93.4 % (95 % CI 91.2; 100.8) at 5 years. Note, however, that the HER2-type subgroup had the second poorest outcome with a LCR of 90.5 % (95 % CI 84.7; 96.3) and a ROS of 88.8 % (95 % CI 81.5; 96.1) (Table 3). The poor survival of triple-negative tumors reflects the lack of effective and specific therapy for this subgroup of patients.

### Strengths and limitations

This study adds recent up-to-date outcomes for the 5 different molecular subtypes from a large prospective cohort with a broad usage of Ki-67 for subtype definition (with a cut-off at 14 %). Unfortunately the subgroups in this study are too small with a favorable outcome and the analysis is underpowered to exhibit further differences in stage distribution as well as surgical and systemic management of primary breast cancer on outcome. The effective disentangling of these and further possible effect would require far larger sample sizes than are present in our data base, and should be pursued within cooperative research projects [40].

In our study, the cohort mirrors the typical, unselected cohort of breast cancer patients treated at a specialized breast cancer unit, as there were no specific exclusion criteria. Unfortunately, we did not systematically document a performance status describing comorbidities. As a prospective, single center study, we cannot exclude any potential center effects that may have confounded the results. Further outcome studies from other clinical settings may help to identify if and to what extent outcomes may vary between different breast units.

## Conclusion

If primary BC is managed at a specialized BCU under guideline-adherent conditions, patients with a favorable subtype can expect an OS above 95 % and an LCR of almost 100 % over 5 years. On the other hand the outcome of patients with HER2 and TN subtypes remains poor, thus necessitating more intensified research and care.

## Additional files

Additional file 1: Table S4. Case frequency for subtypes along UICC stages for patients treated at Heidelberg Breast Care Unit between 01 January 2003, and 31 December 2012). (DOCX 18 kb) Additional file 2: Table S5. Patient characteristics along breast cancer subtypes for patients diagnosed at Heidelberg Breast Care Unit between 01 January 2003 and 31 December 31 2012. (DOCX 17 kb) Additional file 3: Table S6. Five-year Overall Survival exemplary for 3 Subtypes subdivided into UICC stages I and IIa for patients treated at Heidelberg Breast Care Unit between 01 January 2003 and 31 December 2012. (DOCX 15 kb)

## Abbreviations

BC: Breast cancer
BCU: Breast cancer units
CI: confidence interval
CIS: Carcinoma-in-situ
DDFS: Distant disease-free survival
DFS: Disease-free survival
ER: Estrogen receptor
GEP: Gene expression profiles
HER2: Human epidermal growth factor receptor 2
IHC: Immunohistochemistry
LCR: Local control rate
OS: Overall survival
PR: Progesterone receptor
ROS: Relative overall survival
UICC: Union Internationale contre le cancer
