# Supplementary material for: Prognosis of breast cancer molecular subtypes in routine clinical care: A large prospective cohort study
Source: BMC Cancer. 2016 Sep 15;16:734. doi: 10.1186/s12885-016-2766-3 (PMC5024419; doi:10.1186/s12885-016-2766-3)
Supplement: Additional file 1: Table S4. — Case frequency for subtypes along UICC stages for patients treated at Heidelberg Breast Care Unit between 01 January 2003, and 31 December 2012). (DOCX 18 kb) [file 12885_2016_2766_MOESM1_ESM.docx]

| **[%]**  **(n)** | **UICC stage definitions** | | | **LumA-like** | **LumB/HER2 neg.-like** | **LumB/HER2 pos.-like** | **HER2**  **type** | **Triple**  **negative** |
| --- | --- | --- | --- | --- | --- | --- | --- | --- |
| **Stage I**  (n=1501) | T1 | N0 | M0 | 57.8  (n=868) | 41.3  (n=425) | 44.9  (n=80) | 47.1  (n=63) | 40.8  (n=144) |
| **Stadium IIa**  (n=871) | T0,T1 | N1 | M0 | 24.4  (n=365) | 28.6  (n=295) | 28.1  (n=50) | 21.6  (n=29) | 37.4  (n=132) |
|  | T2 | N0 | M0 |  |  |  |  |  |
| **Stage IIb**  (n=278) | T2 | N1 | M0 | 8.1  (n=122) | 9.8  (n=101) | 9.0  (n=16) | 11.2  (n=15) | 6.8  (n=24) |
|  | T3 | N0 | M0 |  |  |  |  |  |
| **Stage IIIa**  (n=263) | T0,T1, T2 | N2 | M0 | 6.3  (n=95) | 10.8  (n=111) | 10.1  (n=2) | 10.5  (n=14) | 7.1  (n=25) |
|  | T3 | N1,N2 | M0 |  |  |  |  |  |
| **Stage IIIb**  (n=47) | T4 | N0,N1,N2 | M0 | 0.9  (n=14) | 2.4  (n=25) | 1.1  (n=2) | 0.8  (n=1) | 1.4  (n=5) |
| **Stage IIIc**  (n=157) | every T | N3 | M0 | 1.5  (n=37) | 7.1  (n=73) | 6.7  (n=12) | 9.6  (n=12) | 6.5  (n=23) |
|  | | | | 100.0 (n=1501) | 100.0 (n=1030) | 100.0  (n=178) | 100.0 (n=134) | 100.0 (n=353) |
|  | | | | **n=3196**  (difference to n=3454 due to missings and pathological complete remission after neoadjuvant therapy) | | | | |
| UICC: Union internationale contre le cancer | | | | | | | | |

**Additional File: Table S4.** Case frequency for subtypes along UICC stages for patients treated at the Heidelberg Breast Care Unit between 01 January 2003 and 31 December 2012.
