# Supplementary material for: Prognosis of breast cancer molecular subtypes in routine clinical care: A large prospective cohort study
Source: BMC Cancer. 2016 Sep 15;16:734. doi: 10.1186/s12885-016-2766-3 (PMC5024419; doi:10.1186/s12885-016-2766-3)
Supplement: Additional file 2: Table S5. — Patient characteristics along breast cancer subtypes for patients diagnosed at Heidelberg Breast Care Unit between 01 January 2003 and 31 December 31 2012. (DOCX 17 kb) [file 12885_2016_2766_MOESM2_ESM.docx]

| **[%]**  **(n)** | **LumA-like** | **LumB/HER2 neg.-like** | **LumB/HER2 pos.-like** | **HER2-type** | **Triple negative** |
| --- | --- | --- | --- | --- | --- |
| **Age at diagnosis in years (n=3454)** | | | | | |
| **median [years]** | 60 | 60 | 57 | 55 | 54 |
| **< 51** | 23.4  (n=361) | 30.7  (n=337) | 40.9  (n=88) | 40.4  (n=69) | 42.2  (n=179) |
| **51-65** | 41.8  (n=654) | 38.4  (n=422) | 37.2  (n=80) | 42.1  (n=72) | 38.9  (n=165) |
| **> 65** | 34.9  (n=539) | 30.9  (n=340) | 21.9  (n=47) | 17.5  (n=30) | 18.9  (n=80) |
| **total** | 100.0  (n=1545) | 100.0  (n=1099) | 100.0  (n=215) | 100.0  (n=171) | 100.0  (n=424) |
| **Menopausal status (n=3454)** | | | | | |
| **pre** | 30.0  (n=463) | 31.5  (n=346) | 36.0  (n=77) | 39  (n=67) | 43.1  (n=183) |
| **peri** | 2.7  (n=42) | 3.5  (n=38) | 3.8  (n=8) | 2.9  (n=5) | 2.7  (n=11) |
| **post** | 65.2  (n=1007) | 62.0  (n=681) | 57.8  (n=124) | 55.9  (n=96) | 52.0  (n=220) |
| **missing** | 2.1  (n=33) | 3.0  (n=34) | 2.4  (n=6) | 2.2  (n=3) | 2.2  (n=10) |
| **total** | 100.0  (n=1545) | 100.0  (n=1099) | 100.0  (n=215) | 100.0  (n=171) | 100.0  (n=424) |
| **Affected breast (n=3454)** | | | | | |
| **left** | 51.2  (n=791) | 51.0  (n=560) | 51.2  (n=110) | 50.7  (n=86) | 50.5  (n=214) |
| **right** | 48.8  (n=754) | 49.0  (n=539) | 48.8  (n=105) | 49.3  (n=85) | 49.5  (n=210) |
| **total** | 100.0  (n=1545) | 100.0  (n=1099) | 100.0  (n=215) | 100.0  (n=171) | 100.0  (n=424) |

**Additional File: Table S5.** Patient characteristics along breast cancer subtypes for patients diagnosed at the Heidelberg Breast Care Unit between 01 January 2003 and 31 December 2012.
