# Supplementary material for: Prognosis of breast cancer molecular subtypes in routine clinical care: A large prospective cohort study
Source: BMC Cancer. 2016 Sep 15;16:734. doi: 10.1186/s12885-016-2766-3 (PMC5024419; doi:10.1186/s12885-016-2766-3)
Supplement: Additional file 3: Table S6. — Five-year Overall Survival exemplary for 3 Subtypes subdivided into UICC stages I and IIa for patients treated at Heidelberg Breast Care Unit between 01 January 2003 and 31 December 2012. (DOCX 15 kb) [file 12885_2016_2766_MOESM3_ESM.docx]

**Additional File:** **Table S6.** Five-year Overall Survival exemplary for 3 Subtypes subdivided into UICC stages I and IIa for patients treated at Heidelberg Breast Care Unit between 01 January 2003 and 31 December 2012.

| **5-year Overall Survival [%] (95% CI)** | **LumA-like** | **LumB/HER2 neg.-like** | **Triple negative** |
| --- | --- | --- | --- |
| **Stage I** | 97.8 [96.5 ; 99.1]  (n=868) | 97.6 [95.7 ; 99.5]  (n=425) | 89.3 [83.5 ; 95.1]  (n=144) |
| **Stage IIa** | 95.4 [92.4 ; 98.4]  (n=365) | 94.9 [91.8 ; 98.0]  (n=295) | 79.0 [70.9 ; 87.1]  (n=122) |
| CI: confidence intervall | | | |
